# Intrathoracic anastomotic leak after esophagojejunostomy in a patient with preoperative coronavirus disease 2019: A case treated with T-tube drainage

**DOI:** 10.1016/j.ijscr.2025.111844

**Published:** 2025-08-19

**Authors:** Takuma Kurotaki, Yuma Ebihara, Hirotake Abe, Hideyuki Wada, Toshiaki Shichinohe, Satoshi Hirano

**Affiliations:** a Department of Gastroenterological Surgery II, Hokkaido University Graduate School of Medicine, Sapporo, Hokkaido, Japan

**Keywords:** Intrathoracic anastomosis, Anastomotic leak, Coronavirus disease 2019, T-tube, Case report

## Abstract

**Introduction:**

Intrathoracic anastomotic leakage can lead to severe complications, such as mediastinitis and empyema, with a high mortality rate. However, the optimal treatment strategy for anastomotic leakage remains controversial.

**Presentation of case:**

This case involves a 55-year-old male patient with esophagogastric junction cancer who experienced intrathoracic anastomotic leakage following esophagojejunostomy, exacerbated by coronavirus disease 2019 (COVID-19)-related immunodeficiency. Following conservative therapy, the patient had a right-sided empyema, necessitating thoracoscopic pleural decortication on postoperative day (POD) 35. A T-tube was inserted at the anastomotic site, and its short limb maintained stability within the lumen, facilitating fistula formation. Postoperatively, the patient's body temperature and inflammatory markers gradually returned to normal. Oral intake was resumed on POD68, and the T-tube was removed on POD79. Subsequently, the patient was transferred to another hospital for rehabilitation on POD96.

**Discussion:**

In this patient with COVID-19 infection and systemic sepsis, T-tube drainage facilitated fistula formation and ensured continuous decompression at the anastomotic site, contributing to successful conservative management.

**Conclusion:**

T-tube drainage may be an effective and feasible treatment option for intrathoracic anastomotic leakage in patients with severe immunodeficiency.

## Highlights

• T-tube drainage may be a feasible option for intrathoracic anastomotic leakage in patients with severe immunodeficiency.
• Adequate drainage is crucial for successful management of intrathoracic anastomotic leakage.
• Preoperative COVID-19 infection may lead to severe respiratory symptoms and adversely affect the postoperative course.

## 1 Introduction

The incidence of esophagogastric junction (EGJ) cancer is increasing worldwide, and intrathoracic anastomosis is typically required for surgical resection. Anastomotic leakage is a severe complication following intrathoracic anastomosis, particularly in the thoracic cavity. However, an optimal treatment strategy for anastomotic leakage remains controversial. Here, we present a case of intrathoracic anastomotic leakage following EGJ cancer surgery in a patient with coronavirus disease 2019 (COVID-19)-related immunodeficiency, successfully managed using T-tube (Create Medic Co. Ltd., Kanagawa, Japan) drainage.

This work has been reported in line with the SCARE criteria [1].

## 2 Presentation of case

A 55-year-old Asian male presented with dysphagia. A previous medical checkup revealed hypertension and cholelithiasis. The patient had a 5-year smoking history of three cigarettes per day and had not received a COVID-19 vaccination.

**Fig. 1:**
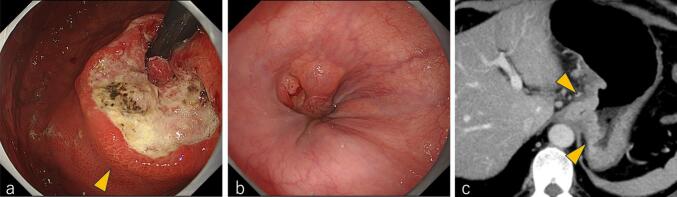
Imaging before chemotherapy. (a) Endoscopic image showing a type 3 tumor (arrowhead). (b) Endoscopy showed a 3 cm esophageal invasion. (c) Contrast-enhanced computed tomography image showing a thickened wall around the EGJ (arrowheads), suggesting tumor invasion of the left crus. EGJ: Esophagogastric junction.

Upper gastrointestinal endoscopy identified a type 3 tumor at the esophagogastric junction, with 3 cm of esophageal invasion (Fig. 1a, b). Contrast-enhanced computed tomography (CT) scan revealed a thickened wall around the EGJ, suggesting tumor invasion into the left crus (Fig. 1c). Biopsy confirmed well-differentiated tubular adenocarcinoma. Clinically, the patient was classified as cT4aN2M0, Stage IVA.

For neoadjuvant chemotherapy (NAC), the patient received three courses of S-1 (Tegafur, Gimeracil, and Oteracil Potassium) + oxaliplatin therapy, which resulted in shrinkage of the primary tumor and associated lymph nodes. Post-NAC, the TNM stage was downstaged to ycT4aN1M0, Stage III. The patient's tumor marker levels were elevated prior to NAC, with a carcinoembryonic antigen (CEA) and carbohydrate antigen 19–9 (CA19–9) level of 9.8 ng/mL and 114.0 U/mL, respectively. Following NAC, the levels of these markers returned to the normal ranges.

A lower esophagectomy and total gastrectomy were performed using a combined laparoscopic and thoracoscopic approach, followed by Roux-en-Y reconstruction with intrathoracic anastomosis and cholecystectomy. The esophagus was transected at the level of the lower pulmonary vein. We performed an intracorporeal circular stapling esophagojejunostomy using a transoral anvil (OrVill; Covidien, Mansfield, MA, USA) and circular stapler (EEA25; Covidien) under indocyanine green fluorescence imaging guidance. The stapler was inserted into the jejunal limb and connected to the anvil to complete the anastomosis under simultaneous laparoscopic and thoracoscopic visualization. Anastomosis was performed via a retrocolic route, and the jejunal stump was closed using a 60-mm linear stapler. The surgical procedure was performed as previously described [2]. A 19Fr silicone drain was placed posterior to the anastomotic site. The final pathological staging was ypT3N0M0, Stage IIA.

**Fig. 2:**
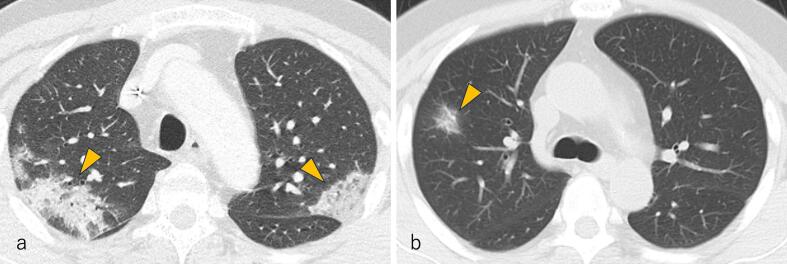
Pre- and postoperative thoracic CT findings. (a) Computed tomography (CT) on postoperative day 5 revealed new ground-glass opacity (GGO) in both lungs(arrowheads). (b) CT performed one day prior to surgery showing a new GGO in the right upper lobe (arrowhead). GGO: Ground-glass opacity CT: Computed tomography.

On postoperative day (POD) 2, the patient had a fever and productive cough, prompting the initiation of antibiotic therapy. On POD5, a routine CT revealed no significant findings, except for new ground-glass opacities (GGO) in both lungs (Fig. 2a). On POD7, a positive severe acute respiratory syndrome coronavirus 2 (SARS-CoV-2) PCR test was observed. The patient's SpO_2_ decreased to 93 % in room air and was subsequently diagnosed with moderate COVID-19 pneumonia. Treatment with dexamethasone (6 mg daily) and remdesivir (5 days) was initiated. The patient was hospitalized 7 days preoperatively and was later found to have been exposed to an asymptomatic individual who tested positive for SARS-CoV-2 on the day of admission. He had experienced a mild fever 4 days before surgery but had no other symptoms. A routine CT performed 1 day preoperatively showed GGO in the right lung (Fig. 2b). At that time, no further investigation was performed, as the fever was self-limited and without respiratory symptoms. In retrospect, both the fever and GGO were likely early signs of COVID-19 pneumonia.

**Fig. 3:**
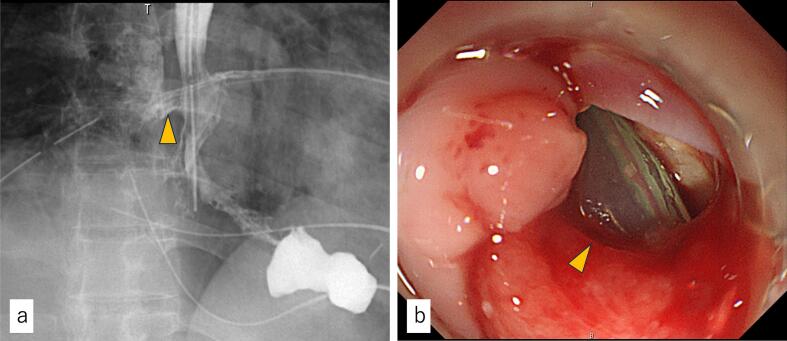
Postoperative image. (a) Oral contrast study on POD13 revealed a leak to the right thoracic space. (arrowhead) (b) Enteroscopy on POD29 showed a one-quarter circumferential anastomotic perforation. A surgical drain located posterior to the anastomotic site was visible through the perforation. POD: Postoperative day.

On POD8, the fluid from the surgical drain became milky white. Fluid analysis revealed an amylase level of 24,000 U/L and a triglyceride level of 1008 mg/dL. Although the high triglyceride level suggested a chylous component, the extremely elevated amylase concentration supported the diagnosis of an anastomotic leak rather than an isolated chyle leak. A gastric tube was inserted and maintained under continuous negative pressure at −12 cmH2O. An initial oral contrast study performed on POD8 showed no evidence of anastomotic leakage, but a follow-up study on POD13 revealed leakage into the thoracic space (Fig. 3a). On POD19, the patient had septic shock secondary to *Candida* bacteremia and required intubation. Antifungal therapy was initiated, and steroid therapy was discontinued. A double-lumen decompression and feeding tube (W-ED tube; Cardinal Health, Dublin, OH, USA) was placed, and enteral nutrition was initiated while decompression was maintained through the tube. Gastroscopy on POD29 revealed a one-quarter circumferential anastomotic perforation (Fig. 3b). Approximately 80 % of the defect was closed using the Mantis™ Closure Device (Boston Scientific, Marlborough, MA, USA). Three days later, a follow-up gastroscopy revealed ulceration around the clips. Nasoesophageal extraluminal drainage (NEED) was initiated on POD32, and decompression was continued through the tube.

**Fig. 4:**
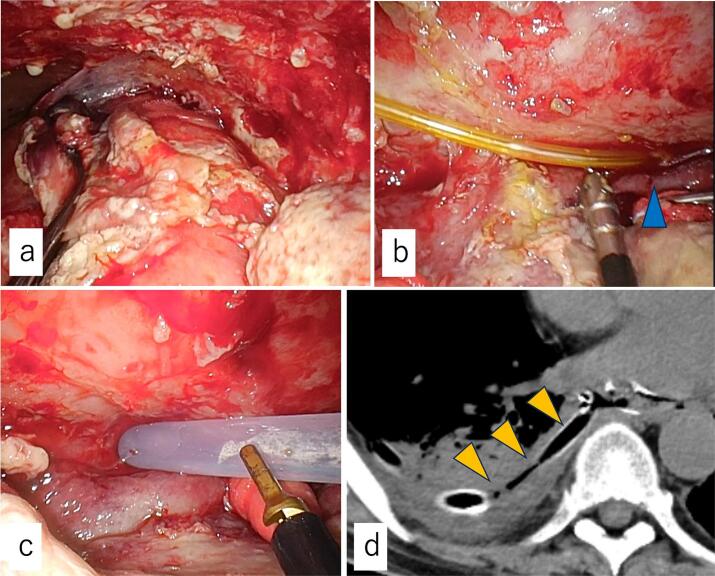
Operative findings. (a) Abundant fibrinous deposits observed in the right pleura. (b) The NEED tube was located in the right thoracic cavity via the anastomotic site (arrowhead). (c) A T-tube is inserted at the anastomotic site. (d) Postoperative computed tomography showed that the T-tube (arrowheads) positioned posterior to the lung. NEED: Nasoesophageal extraluminal drainage.

The patient remained febrile, and CT revealed fluid accumulation in the right thoracic cavity, leading to a diagnosis of right-sided empyema. On POD35, a tracheotomy and thoracoscopic lung decortication were performed. The tracheotomy was performed to secure the airway, as the patient required prolonged respiratory support due to septic shock and right-sided empyema. Abundant fibrinous deposits were observed in the right pleura (Fig. 4a). A NEED drain was placed in the right thoracic cavity at the anastomotic site (Fig. 4b). Subsequently, the NEED tube was removed, and a T-tube was inserted intraoperatively at the anastomotic site (Fig. 4c) after the short limb was cut in a half-round manner. Each side of the short limb was trimmed to a length of 2 cm, and the T-tube was positioned posterior to the lung (Fig. 4d). Postoperatively, the patient's body temperature and inflammatory markers gradually returned to normal. On POD53, respiratory support was discontinued. An oral contrast study on POD68 showed no leakage into the T-tube, and oral intake was subsequently resumed. On POD79, the T-tube migrated into the fistula tract, and a follow-up contrast-enhanced CT showed no evidence of anastomotic leakage. After five days of fasting, oral intake was restarted, and the patient was transferred for rehabilitation on POD96.

## 3 Discussion

The incidence of EGJ cancer has been rising in Asia and Europe [3], largely due to increasing obesity, gastroesophageal reflux disease, and declining *Helicobacter pylori* infection [4]. Anastomosis is commonly performed in the thoracic cavity for patients with EGJ cancer. Intrathoracic anastomotic leakage can lead to severe complications such as mediastinitis and empyema, with high mortality rates of 9.3–12.1 % [5,6]. Inadequate drainage of the abscess cavity increases the risk of empyema [5], potentially leading to treatment failure and prolonged hospitalization. The thoracic cavity contains less fatty tissue than the abdomen, which may delay fistula formation and prolong healing. Therefore, ensuring adequate drainage and promoting early fistula formation are crucial for successful treatment.

Treatment for intrathoracic leakage includes conservative therapy with drainage, stenting, and endoscopic clipping; direct suturing; or re-anastomosis [7]. Pleural decortication is required if empyema is present. However, the optimal treatment strategy remains elusive as the choice of treatment depends on multiple factors such as the size of the defect, infection status, and patient condition.

The patient was immunocompromised due to COVID-19 infection and *Candida* sepsis, increasing the risk of anastomotic leakage; therefore, direct suturing or re-anastomosis was not considered feasible in this case. Given these factors, we opted for endoscopic closure as a viable treatment option. Although we attempted to seal the perforation with a MANTIS device, the defect was too large for complete closure. Stenting remains an alternative but carries risks of post-placement complications.

Adequate drainage is a cornerstone of conservative therapy for anastomotic leakage. The three-drain method, which includes feeding, gastrointestinal decompression, and chest drainage tubes [8], is an efficient approach. Yin et al. reported that NEED, which places a drain through the esophagus into the abscess cavity, results in earlier healing than conventional drainage from the chest wall [8]. In this case, NEED was performed postoperatively.

During pleural decortication surgery, we inserted a T-tube to promote fistula formation. Although the T-tube is well-established for Boerhaave syndrome, only two studies have specifically reported its application [9,10] to anastomotic leaks. We did not suture the T-tube in place but guided it along the dorsal side of the lung to encourage fistula formation. The T-tube has a short limb that remains stably positioned within its lumen. Its soft, flexible design protects adjacent tissues and promotes decompression at the anastomosis site. In our patient, who had a systemic infection beginning with COVID-19 infection, adequate drainage was achieved with a T-tube, and the anastomotic leakage subsequently healed. NEED was performed three days before the reoperation but was removed intraoperatively, precluding efficacy assessment. Continuing NEED after pleural decortication surgery could have been viable; however, we selected the T-tube drainage to allow better long-term drainage and to promote fistula formation. This experience underscores the T-tube's value in complex, high-risk cases and supports individualized management strategies.

The relationship between COVID-19 and anastomotic leakage remains unclear; however, COVID-19 is well-known to induce cytokine storms and endothelial dysfunction [11]. Therefore, an association between COVID-19 and anastomotic leakage cannot be ruled out. An analysis using the OpenSAFELY platform in the United Kingdom revealed that the postoperative mortality rate was 4.1 % among patients who underwent surgery within 14 days post-COVID-19 infection between May 2018 and January 2021, compared to 1.1 % between January 2021 and March 2022, when vaccination had become widely used [12]. The patient in the present case had not received a COVID-19 vaccination and experienced respiratory failure. Furthermore, the risk of adverse outcomes declines seven weeks after the onset of COVID-19 infection [12].

The patient's prolonged postoperative course was influenced by COVID-19 infection, Candida sepsis, and empyema, which delayed healing and necessitated extended intensive care. This case underscores the need for thorough preoperative assessment and careful surgical indication in patients with suspected infections. Early identification and management of infectious risk factors may reduce postoperative complications and improve outcomes.

## 4 Conclusion

Intrathoracic anastomotic leakage can lead to severe complications, such as mediastinitis and empyema, with a high mortality rate. We experienced a rare case of preoperative COVID-19 infection, which resulted in anastomotic leakage and systemic infection. Adequate drainage was achieved with a T-tube, and the anastomotic leakage subsequently healed. Therefore, T-tube drainage may be an effective and feasible treatment option for intrathoracic anastomotic leakage in patients with severe immunodeficiency.

## Consent for publication

Written informed consent for publication of this article and accompanying images was obtained from the patient.

## Ethical approval

This case report involved a single patient and no research intervention. According to common institutional practice in Japan, ethics approval is not required in such cases.

## Guarantor

Takuma Kurotaki.

## Funding

All authors have no funding.

## Author contribution

Takuma Kurotaki wrote the manuscript. Yuma Ebihara, Hideyuki Wada, and Satoshi Hirano revised the manuscript. Yuma Ebihara, Hirotake Abe, Toshiaki Shichinohe, and Satoshi Hirano contributed to the treatment of the patient. All authors have read and approved the final version of the manuscript and share full responsibility for its content.

## Declaration of competing interest

None.

## Data Availability

Not applicable.
